# Constitutive expression of cathepsin K in the human intervertebral disc: new insight into disc extracellular matrix remodeling via cathepsin K and receptor activator of nuclear factor-κB ligand

**DOI:** 10.1186/ar3454

**Published:** 2011-08-31

**Authors:** Helen E Gruber, Jane A Ingram, Gretchen L Hoelscher, Natalia Zinchenko, H James Norton, Edward N Hanley

**Affiliations:** 1 Department of Orthopaedic Surgery, Carolinas Medical Center, PO Box 32861, Charlotte, NC 28232, USA; 2 Department of Biostatistics, Carolinas Medical Center, PO Box 32861, Charlotte, NC 28232, USA

## Abstract

**Introduction:**

Cathepsin K is a recently discovered cysteine protease which cleaves the triple helical domains of type I to II collagen. It has been shown to be up-regulated in synovial tissue from osteoarthritic and rheumatoid patients, and is a component in normal and nonarthritic cartilage, where it increases with aging. Studies on heart valve development have recently shown that receptor activator of nuclear factor-κB ligand (RANKL) acts during valve remodeling to promote cathepsin K expression. Since extracellular matrix remodeling is a critical component of disc structure and biomechanical function, we hypothesized that cathepsin K and RANKL may be present in the human intervertebral disc.

**Methods:**

Studies were performed following approval of the authors' Human Subjects Institutional Review Board. Six annulus specimens from healthier Thompson grade I to II discs, and 12 specimens from more degenerate grade III to IV discs were utilized in microarray analysis of RANKL and cathepsin K gene expression. Immunohistochemistry was also performed on 15 additional disc specimens to assess the presence of RANKL and cathepsin K.

**Results:**

Cathepsin K gene expression was significantly greater in more degenerated grade III to IV discs compared to healthier grade I to II discs (*P *= 0.001). RANKL was also identified with immunohistochemistry and molecular analyses. RANKL gene expression was also significantly greater in more degenerated discs compared to healthier ones (*P *= 0.0001). A significant linear positive correlation was identified between expression of cathepsin K and RANKL (r^2 ^= 92.2; *P *< 0.0001).

**Conclusions:**

Extracellular matrix remodeling is a key element of disc biology. Our use of an appropriate antibody and gene expression studies showed that cathepsin K is indeed present in the human intervertebral disc. Immunolocalization and molecular analyses also confirmed that RANKL is present in the human disc. Expression of RANKL was found to be significantly greater in more degenerated compared to healthier discs (*P *= 0.0001). Cathepsin K gene expression levels showed a positive, significant correlation with RANKL expression. Based on these data, we propose that cathepsin K plays a significant role in disc matrix remodeling and in matrix degradation in the proinflammatory cytokine-rich microenvironment of the degenerating disc.

## Introduction

Cathepsin K, discovered and isolated from a rabbit osteoclast library in 1994 [1], is an interesting cysteine protease which cleaves the triple helical domains of types I to II collagen [1]. Research has shown that its collagenolytic action requires that chondroitin or keratin sulfate be bound to the protease [2]. Cathepsin K is present in osteoclasts, human ovary, heart and skeletal muscle, lung, placenta, testis, small intestine and colon [3]. Studies have identified upregulation of cathepsin K in fibroblast-like cells in synovial tissue of osteoarthritic and rheumatoid patients [4-7]. A link to heightened expression in the presence of IL-1ß or TNFα was also shown by *in vitro *culture of fibroblasts derived from rheumatoid patients in the work of Hou *et al*. [5]. In addition, an association with aging and increased cathepsin K levels has been suggested by Dejica *et al*. since they found increased cathepsin K content in nonarthritic cartilage from older compared to younger subjects [8]. Work by Ruettger *et al*. showed that cathepsin K is regulated via activation of the classical protein kinase C and p38 MAP kinase in articular chondrocytes [9].

Two interesting reports have shown spontaneous development of synovitis and cartilage degeneration in transgenic mice, which overexpress cathepsin K (transgenic UTU17 mice) [10,11]. In analysis of this model, transgenic animals showed osteoarthritis and increased levels of cathepsin K with aging. During growth and aging, cathepsin K was found to be the most abundant cysteine proteinase in the mouse knee joint, where cathepsin K was present near sites of matrix degeneration and destruction in articular cartilage.

Cathepsin K is a recognized component of osteoclasts, where it plays a central role in bone resorption [7]. RANKL (receptor activator of nuclear factor κB ligand, also known as TRANCE, TNFSF11, OPGL, and ODF) is a member of the tumor necrosis factor family of signaling molecules, which functions in promoting osteoclast differentiation. RANKL, well known for its role in production of extracellular matrix remodeling enzymes, is one of the factors capable of inducing cathepsin K production. This aspect of the biology of cathepsin K has recently been studied by Combs and Yutzey in an analysis of the regulation of heart valve development which found that RANKL and cathepsin K are expressed by endocardial cushion endothelial cells; RANKL acted during valve remodeling to promote cathepsin K expression [12].

Although there is not a large body of literature addressing cathepsin K and its role in disc degeneration, there are several previous interesting studies. The work of Ariga *et al*. pointed to an association with endplate separation and disorganization of the annulus in spinal degenerative disorders [13]. Neidhart *et al*. found strong expression of cathepsin K in a number of regions of the spine in patients with ankylosing spondylitis [14]. Mwale *et al*. have studied the effect of a collagen type II collagen fragment (the 245-270 peptide) on disc cells [15]. This work showed that the addition of this peptide fragment at levels of 1 μg/ml to cultured annulus cells resulted in a significant increase in cathepsin K expression by one day of culture; expression levels decreased over the following four days of culture and reached control levels. Nucleus cells exposed to this fragment also showed stimulated cathepsin K expression at one day of exposure.

In the present study, we hypothesized that cathepsin K might be an important component of matrix remodeling overlooked to date in the intervertebral disc. We tested this hypothesis by assessing gene expression of cathepsin K and RANKL in human disc tissue, and also applied immunohistochemistry analyses to disc tissues.

## Materials and methods

### Clinical study population

Experimental study of human disc specimens was approved prospectively by the authors' Human Subjects Institutional Review Board at Carolinas Medical Center. The need for informed consent was waived by the ethical board since disc tissue was removed as part of routine surgical practice. Scoring of disc degeneration utilized the Thompson scoring system; this system scores disc degeneration over the spectrum from a healthy disc (Thompson grade I) to discs with advanced degeneration (grade V, the most advanced stage of degeneration) [16]. Patient specimens were derived from surgical disc procedures performed on individuals with herniated discs and degenerative disc disease. Surgical specimens were transported to the laboratory in sterile tissue culture medium. Care was taken to remove all granulation tissue and to sample only disc tissue. Non-surgical control donor disc specimens were obtained via the National Cancer Institute Cooperative Human Tissue Network (CHTN); they were shipped overnight to the laboratory in sterile tissue culture medium and processed as described below. Specimen procurement from the CHTN was included in our approved protocol by our human subjects Institutional Review board.

### Expression of cathepsin K and RANKL *in vivo*

Analysis of human disc tissue was carried out as previously described using laser capture microdissection methods [17]. Total RNA was extracted from cells using the TRIzol reagent (Gibco, Carlsbad, CA, USA), reverse transcribed to double-stranded cDNA, subjected to two rounds of transcription, and hybridized to the DNA microarray in the Affymetrix Fluidics Station 400. Affymetrix human U133 X3P arrays were used. The GCOS Affymetrix GeneChip Operating System (version 1.2, Affymetrix, Santa Clara, CA, USA) was used for determining gene expression levels of RANKL and cathepsin K (Affymetrix gene identifications AF053712.1 for RANKL, and NM_000396.1 for cathepsin K).

Gene array data related to human disc tissue reported here have been uploaded to the Gene Expression Omnibus (GEO) website [GEO:GSE23130] and may be accessed at sample numbers GSM569830 - GSM569848.

### Immunolocalization of RANKL

Disc specimens were fixed in 10% neutral buffered formalin for no longer than 24 hours and changed to 70% ethanol. Undecalcified specimens were embedded in paraffin and sections cut at 4 μm, collected on PLUS slides (Allegiance, McGaw Park, IL, USA) and dried at 60°C. Sections were deparaffinized in xylene (Allegiance) and rehydrated through graded alcohols (AAPER, Shelbyville, KY, USA) to distilled water. Antigen retrieval was performed using Dako Target Retrieval Solution, pH 6.0 (Dako, Carpenteria, CA, USA) for 20 minutes at 95°C followed by cooling for 20 minutes. The remainder of the procedure was performed using the Dako Autostainer Plus (Dako) automated stainer. Endogenous peroxidase was blocked using 3% H_2_0_2 _(Humco, Texarcana, TX, USA). Slides were incubated for one hour with anti-RANKL (Santa Cruz Biotechnology, Santa Cruz, CA, USA) at a 1:50 dilution. Secondary antibody was 4+Biotinylated Universal Goat Link (Biocare Medical, Concord, CA, USA) for 10 minutes followed by 4+ streptavidin HRP Label (Biocare) for 10 minutes and Vector NovaRed (Vector Laboratories, Burlingame, CA, USA) for 5 minutes. Slides were removed from the stainer, rinsed in water, counterstained with light green, dehydrated, cleared and mounted with resinous mounting media. Mouse IgG (Dako) was used as a negative control. Human tonsil was utilized as a positive control.

### Immunolocalization of cathepsin K

Following fixation and embedding as described above, paraffin sections were cut at 4 μm, collected on PLUS slides (Allegiance) and dried at 60°C. Sections were deparaffinized in xylene (Allegiance) and rehydrated through graded alcohols (AAPER) to distilled water. The remainder of the procedure was performed using the Dako Autostainer Plus (Dako) automated stainer. Endogenous peroxidase was blocked using 3% H_2_0_2 _(Humco). Slides were incubated for one hour with anti-cathepsin K (Abcam, Cambridge, MA, USA) at a 1:100 dilution. Mouse IgG (Dako) was used as a negative control. A secondary antibody was 4+Biotinylated Universal Goat Link (Biocare Medical,) for 10 minutes followed by 4+ streptavidin HRP Label (Biocare) for 10 minutes and DAB (Biocare)) for 5 minutes. Slides were removed from the stainer, rinsed in water, counterstained with light green, dehydrated, cleared and mounted with resinous mounting media. Rat physeal tissue was used as a positive control.

### Statistical analyses

Standard statistical analyses were performed utilizing InStat (GraphPad Software, Inc., San Diego, CA, USA). Unpaired t-tests and linear regression analyses were performed; means ± s.e.m. were calculated. *P *= 0.05 was considered to be the level of significant.

## Results

### Molecular confirmation of *in vivo *expression of Cathepsin k and RANKL expression in the human annulus

**Figure 1:**
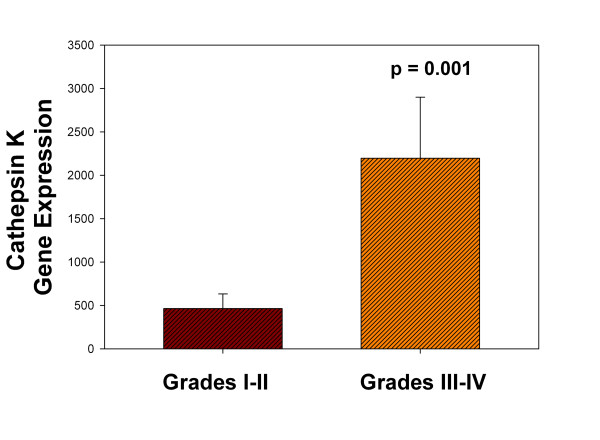
**Cathepsin K expression and stages of disc degeneration**. Cathepsin K gene expression was significantly greater in annulus tissue from more degenerated discs than in healthier discs (*P *= 0.001). Data are means ± s.e.m.

**Figure 2:**
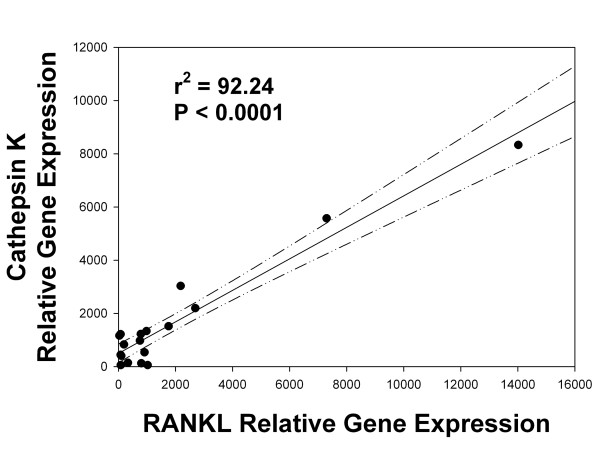
**Relationship between RANKL expression and cathepsin K expression**. A significant, positive correlation was present between gene expression levels of RANKL and cathepsin K (r^2 ^= 92.2; *P *< 0.0001). (Dashed line shows the 95% confidence interval for the correlation).

**Figure 3:**
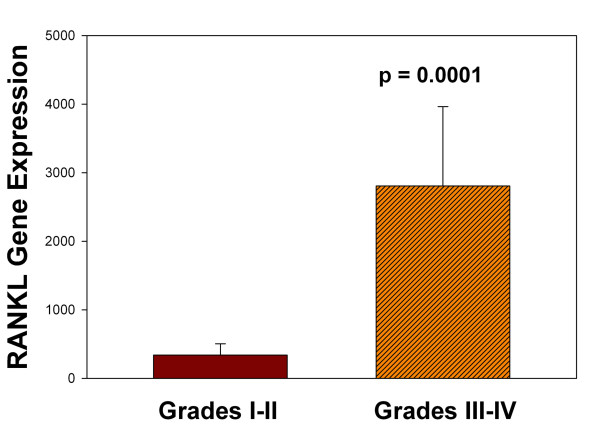
**RANKL expression and stages of disc degeneration**. RANKL gene expression was significantly greater in annulus tissue from more degenerated discs than in healthier discs (*P *= 0.0001). Data are means ± s.e.m.

Six annulus specimens from healthier Thompson grade I to II discs, and 13 specimens from more degenerate grade III to IV discs were utilized in microarray analyses of RANKL and cathepsin K gene expression. Demographic features for this study population are listed in Table 1. As shown in Figure 1, cathepsin K expression was significantly greater in more degenerate Thompson grade III to IV discs than in healthier grade I to II discs (*P *= 0.001). Statistical evaluation also found a positive, significant correlation between the expression levels of cathepsin K and those of RANKL (Figure 2; r^2 ^= 92.2; *P *< 0.0001). RANKL gene expression was also found to be significantly greater in more degenerate Thompson grade III to IV discs compared to healthier grade I to II discs (Figure 3, *P *= 0.0001). Statistical analysis showed that there was no relationship between age and either cathepsin K or RANKL gene expression levels.

**Table 1:** Demographic features for specimens studied for immunocytochemical localization of cathepsin K and RANKL

Subject number	Site	Thompson score	Age/gender	Other information (cause of death)
Annulus specimens				
1	L4 to 5	1.5	45/F	CHTN (unknown)
1	L3 to 4	II	45/F	CHTN (unknown)
2	L5 to S1	II	21/M	Surgical specimen
3	L4 to 5	2.5	40/M	CHTN (MI)
4	L1 to 2	III	33/F	CHTN (PE)
5	L4 to 5	III	33/F	Surgical specimen
6	L3 to 4	III	46/F	Surgical specimen
7	L5 to S1	III	53/M	Surgical specimen
8	L4 to 5	III	29/F	Surgical specimen
9	L2 to 3	III	54/M	Surgical specimen
4	L3 to 4	IV	33/F	CHTN (PE)
10	L3 to 4	IV	59/F	Surgical specimen
10	L1 to 2	IV	59/F	Surgical specimen
10	L2 to 3	IV	59/F	Surgical specimen
11	L3 to 4	IV	78/M	Surgical specimen
12	L2 to 3	IV	56/F	Surgical specimen
13	L4 to 5	IV	39/F	Surgical specimen
14	L5 to S1	V	44/F	Surgical specimen
15	L5 to S1	V	39/F	Surgical specimen
Nucleus specimens				
16	L4 to 5	3.5	68/F	CHTN (stroke)
17	L3 to 4	V	69/M	CHTN (MI)
18	L5 to S1	III	54/F	CHTN (unknown)
19	L2 to 3; L3 to 4	II	45/F	CHTN (unknown)
20	L3 to 4; T12 to L1	IV; 3.5	33/F	CHTN (PE)

CHTN, Cooperative Human Tissue Network; F, female; age is presented in years; L, Lumbar; M, male; MI, myocardial infarction; PE, pulmonary embolism; S, sacral; T, thoracic.

### *In vivo *immunolocalization of cathepsin K and RANKL in the human disc

**Figure 4:**
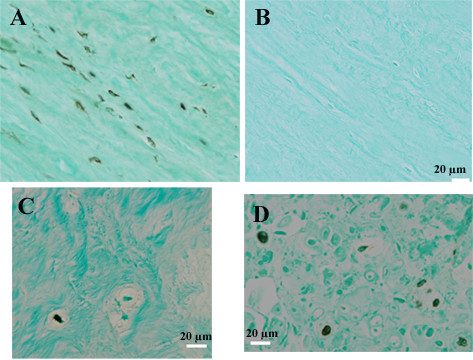
**Representative images showing immunohistochemical localization of cathepsin K in the human disc**. Immunohistochemical localization (black localization product) shows the presence of cathepsin K in the outer annulus (Figure 4A). Figure 4B shows an adjacent negative control section from the outer annulus. Within the inner annulus (Figure 4C) and nucleus (Figure 4D), both positive and negative cells were present. (Scale bar for Figure 4A is the same as that shown for Figure 4B).

Immunohistochemical studies were performed on specimens derived from subjects whose demographic features are described in Table 1. Cells in the outer annulus showed strong immunolocalization of cathepsin K (Figure 4A). Inner annulus and nucleus regions showed that some, but not all, cells were positive for cathepsin K immunolocalization (Figure 4C, D). In the inner annulus, cells in clusters also showed that not all cells were positive for localization.

**Figure 5:**
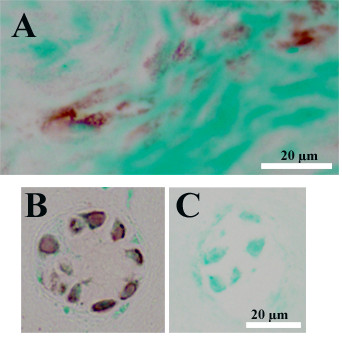
**Representative images showing immunohistochemical localization of RANKL in the human disc**. Immunohistochemical localization of RANKL (red localization product) using antigen retrieval in the outer annulus (Figure 5A) and inner annulus (Figure 5B). Figure 5C shows an adjacent negative control.

RANKL immunohistochemical analyses required antigen retrieval during the localization procedure. Figure 5 shows cells positive for localization in the outer annulus (Figure 5A), and in cells present in clusters in the inner annulus (Figure 5B). Occasional positive cells were identified in the nucleus (data not shown).

## Discussion

Improved understanding of the regulation of extracellular matrix turnover during normal homeostasis and during advancing disc degeneration is an important topic in disc research [18]. As noted by Millward-Sadler *et al*., matrix turnover has implications for the pathogenesis of human disc degeneration [19]. Historically, the matrix metalloproteinases, including MMP-1, -2, -3, -7, -8, -9, -13, -19 and -28 [20-31], have been considered the key players in disc matrix destruction during disc degeneration.

In the present work we confirm that cathepsin K is constitutively expressed in the human disc. Our analysis showed that there was significantly greater expression of cathepsin K in degenerated discs compared to healthier ones (Figure 1). This finding suggests a role for cathepsin K in disc degeneration, and is in agreement with the findings of Konttinen *et al*. that cathepsin K expression increased with the severity of osteoarthritis [6].

We now know that the degenerating disc has increased expression of a number of important genes related to the extracellular matrix [32] and proteoglycans [33], inflammatory cytokines, and matrix-degrading agents, neurotrophins, and cytokines (for reviews and studies see [26,33-41]). *In vitro *studies have been especially helpful in advancing our understanding of inflammatory cytokine production [42-45]. Hou *et al*. have shown that IL-1ß or TNFα *in vitro *stimulation of synovial fibroblasts derived from rheumatoid or osteoarthritic subjects produced increased cathepsin K expression. These two proinflammatory cytokines have well-recognized roles in intervertebral disc degeneration [46]. We hypothesize that in the cytokine rich milieu of the degenerating disc cathepsin K plays a significant role in disc matrix degeneration.

RANKL is recognized as a factor capable of inducing cathepsin K production, and is known to be regulated by IL-1ß and TNFα (see [7] for a recent review). In addition to its acknowledged role in osteoclast development and function [7], RANKL signaling activates a number of pathways important in a number of cell types including bone marrow stromal cells, fibroblasts, mammary endothelial cells, epithelial cells, osteoblasts, osteoclasts and T lymphocytes (see [47,48] for reviews). In 2009, Mackiewicz *et al*. showed the immunohistochemical presence of RANKL in human annulus tissue [49].

In the present work, we identified significantly increased RANKL gene expression in more degenerated discs compared to healthier discs (Figure 3). Our analyses of gene expression data from disc tissue also showed a significant, positive linear relationship between cathepsin K and RANKL expression which accounted for a high proportion of the variability in this relationship (92.2%, Figure 2). This finding is also consistent with regulation of cathepsin K expression during disc degeneration; we note, however, that future mechanistic studies should be undertaken to further explore this control mechanism. Other aspects of RANKL function in the disc merit future studies to determine whether it acts to inhibit proliferation and to induce apoptosis as was seen by McGonigle *et al*. in endothelial cells [50].

It should be noted that the present analyses utilized only annulus cells in the microarray expression studies; we currently are adding nucleus specimens so that future work can explore expression patterns in nucleus cells as well as the annulus. We look forward to data from other disc research labs on this topic. Important future studies should include *in vivo *analyses to determine the exact relationship between cathepsin K and RANKL expression, correlations of cathepsin K levels to collagen fragments within the disc (as pioneered in the important work of Neidart *et al*. [14] and Mwale *et al*. [15], and expanded *in vitro *studies with annulus and nucleus cells.

In closing, a note should also be made concerning the current clinical interest in development of cathepsin K inhibitors because of their ability to inhibit bone resorption [3,7,51,52]. As we continue to move towards the application of biologic therapies for human disc degeneration, it may be efficacious to consider inclusion of selective, specific approaches to inhibit cathepsin K. Yasuca *et al*. reported in 2005 that some cathepsin K inhibitors are now in clinical trials for osteoporosis therapy, and noted that cathepsin K is a preferable drug target for non-inflammatory osteoarthritis with positive pre-clinical data [3].

## Conclusions

Collectively, our results demonstrate the constitutive expression of cathepsin K and RANKL within human intervertebral disc tissue. Gene expression studies showed that both cathepsin K and RANKL expression levels are significantly greater in annulus tissue from more degenerated discs compared to levels in present healthier discs (*P *= 0.001 and 0.0001, respectively). A positive significant correlation was identified between expression levels of cathepsin K and RANKL (r^2 ^= 92.2; *P *< 0.0001). Based on these data, we suggest that cathepsin K may play a significant role in disc matrix remodeling and in matrix degradation in the proinflammatory cytokine-rich microenvironment of the degenerating disc.

## Abbreviations

CHTN: Cooperative Human Tissue Network; GEO: Gene Expression Omnibus; IL-1ß: interleukin-1-beta; MMP: matrix metalloproteinase; p38 MAP kinase: p38 mitogen-activated protein kinase; RANKL: receptor activator of nuclear factor-κ-B ligand; TNFα: tumor necrosis factor-alpha.

## Competing interests

The authors declare that they have no competing interests.

## Authors' contributions

HEG, in collaboration with ENH, conceived and planned the study. GLH performed microarray analyses, while JAI and NZ performed histology and immunohistochemistry. HEG wrote the manuscript and performed statistical analyses with HJN. Co-authors agreed with the finalized submission. All authors read and approved the final manuscript.
